# Autonomic function and inflammation in pregnant women participating in a randomized controlled study of Mindfulness Based Childbirth and Parenting

**DOI:** 10.1186/s12884-023-05528-2

**Published:** 2023-04-10

**Authors:** Lina Rådmark, Walter Osika, Martin Benka Wallén, Eva Nissen, Gunilla Lönnberg, Richard Bränström, Eva Henje, Renee Gardner, Emma Fransson, Håkan Karlsson, Maria Niemi

**Affiliations:** 1 Department of Clinical Neuroscience, Karolinska Institutet, Tomtebodavägen 18A, 171 77 Stockholm, Sweden; 2 Center for Social Sustainability, Department of Neurobiology, Care Sciences and Society, CSS, Center for Social Sustainability, Karolinska Institutet, Alfred Nobels allé 23, 141 83 Huddinge, Sweden; 3 Northern Stockholm Psychiatry, Stockholm Health Care Services, Vårdvägen 1, 112 81 Stockholm, Sweden; 4 Stockholm Health Care Services, Stockholm County Council, Stockholm, Sweden; 5 Karolinska Institutet, Department Women’s and Children’s Health, Stockholm, Sweden; 6 Department of Public Health and Caring Sciences, Uppsala University, Husargatan 3, 752 37 Uppsala, Sweden; 7 Department of Clinical Science, Child and Adolescent Psychiatry, Umeå University, 901 85 Umeå, Sweden; 8 Department of Global Public Health, Karolinska Institutet, Solnavägen 1 E, 10431 Stockholm, Sweden; 9 Department of Microbiology, Tumor and Cell Biology, Karolinska Institutet, Solnavägen 9, 171 65 Stockholm, Sweden; 10 Department of Women´s and Children´s Health, Uppsala University, Akademiska sjukhuset, 751 85 Uppsala, Sweden; 11 Department of Neuroscience, Karolinska Institutet, Stockholm, Sweden

**Keywords:** Mindfulness, Pregnancy, Depression, Inflammation, Heart rate variability

## Abstract

**Background:**

Pregnancy and childbirth are significant events in many women’s lives, and the prevalence of depressive symptoms increases during this vulnerable period. Apart from well documented cognitive, affective, and somatic symptoms, stress and depression are associated with physiological changes, such as reduced heart-rate variability (HRV) and activation of the inflammatory response system. Mindfulness Based Interventions may potentially have an effect on both HRV, inflammatory biomarkers, and self-assessed mental health. Therefore, the aim of this study was to assess the effects of a Mindfulness Childbirth and Parenting (MBCP) intervention on HRV, serum inflammatory marker levels, through an RCT study design with an active control group.

**Methods:**

This study is a sub-study of a larger RCT, where significant intervention effects were found on perinatal depression (PND) and perceived stress. Participants were recruited through eight maternity health clinics in Stockholm, Sweden. In this sub-study, we included altogether 80 women with increased risk for PND, and blood samples and HRV measures were available from 60 of the participants (26 in the intervention and 34 in the control group).

**Results:**

Participants who received MBCP reported a significantly larger reduction in perceived stress and a significantly larger increase in mindfulness, compared to participants who received the active control treatment. However, in this sub-study, the intervention had no significant effect on PND, inflammatory serum markers or measures of HRV.

**Conclusions:**

No significant differences were found regarding changes in HRV measures and biomarkers of inflammation, larger studies may be needed in the future.

**Trial registration:**

ClinicalTrials.gov ID: NCT02441595. Registered 12 May 2015 - Retrospectively registered.

## Background

Pregnancy and childbirth are significant events in many women’s lives, that often entail abrupt lifestyle changes in addition to hormonal and physiological alterations [10]. The prevalence of mental health problems, especially depressive symptoms, is increased during this vulnerable time of life. The rate of women who suffer from depressive disorders has been reported to be 10% during pregnancy and 13% postpartum (World Health Organization [49]). In addition to the mental health impact on women, depression during pregnancy and in the postpartum period – together referred to as perinatal depression (PND) – also affects the health and wellbeing of the child [25]. Despite its well-documented health consequences, PND often remains undetected and untreated [17].

Exposure to stressful life events [42] and stress in everyday life, such as work-related stress [16] has been found to increase the risk of PND. Indeed, life stress overall has been found to incur a medium to strong risk for PND [26].

Apart from an array of well documented cognitive, affective, and somatic symptoms (WHO [48]) depression as well as stress are associated with physiological changes, such as reduced heart-rate variability (HRV) and activation of the inflammatory response system [27, 35, 36]. Psychophysiological research has shown that HRV can be used to index cardiac vagal tone, which represents the contribution of the parasympathetic nervous system to cardiac regulation. HRV can be impacted by self-regulation at the cognitive, emotional, social, and health levels [30].

Importantly, reduced HRV in pregnant women with depression has been related to abnormal uterine artery blood-flow and lower infant birth weight [45]. Also, pregnant mothers with previous anxiety disorders have been shown to have reduced HRV [7, 21, 45].

During pregnancy, the woman’s body undergoes extensive changes in immune system adaptation and inflammatory response, which can be detected through blood samples [8, 29]. Complex adaptations are made to protect the fetus against pathogens as well as to adjust the mother´s immunological response in order to prevent rejection of the fetus, which lead to increases in a number of inflammatory markers during the progression of pregnancy [8, 29]. Moreover, inflammation has been reported to increase the risk of depression and stress-related disorders [14, 31, 37], as well as predict recurrent major depression episodes [51]. Negative maternal emotions in childbirth have been associated with an increase in pro-inflammatory cytokines of both the mother and the infant [22], even if no single biomarker for PND has been identified to date [9, 39].

In recent decades, the use of Mindfulness Based Interventions (MBIs) in clinical settings, and research regarding their effects on various health-related outcomes have increased rapidly. Mindfulness practice was initially secularized and brought to clinical settings by Jon Kabat-Zinn with the Mindfulness Based Stress Reduction program (MBSR) [44]. Taken together, the use of MBIs is wide spread, and the preliminary research in this field shows that antenatal MBIs are feasible and appreciated, and systematic reviews motivate further research in a field that shows promising results [4, 18, 46].

Some of the MBIs have been tailored specifically for pregnant women and their partners and assessed in terms of a variety of outcomes in iterated pilot studies, with only a few with a randomised controlled design and an active control condition [34]. A recently published systematic review [3] and a meta-analysis [18] have concluded that there is sufficient evidence for the use of mindfulness interventions for anxiety, stress and depression reduction in the perinatal period, but that research of the effects on other pregnancy-related outcomes is yet insufficient.

Some studies have documented physiological effects of mindfulness on immune function [13, 15], and on HRV [38], However, a recent meta-analysis on mindfulness based interventions on biomarkers showed mixed and inconclusive results, particularly due to the lack of large, rigorously conducted RCTs [41].

In summary, MBIs may potentially have an effect on HRV and inflammatory biomarkers, and our previous study showed significant effects on PND and perceived stress. Therefore, the aim of this study was to assess the effects of a Mindfulness Based Childbirth and Parenting (MBCP) intervention on HRV and serum inflammatory marker levels, through an RCT study design with an active control group. The active control consisted of a Lamaze childbirth program. This method has been developed initially by Dr Fernand Lamaze [40], and is widely used as childbirth preparation in the Swedish context, where the present study was conducted. The method includes focused breathing techniques, muscle relaxation, and focus practices, and has been shown in a recent meta-analysis to carry positive effects on the length of labor, alleviate labor pain and reduce post-partum bleeding [50]. However, the aim of Lamaze is mainly to assist women in the physical side of labor and does not focus particularly on the common psychological challenges of pregnancy and childbirth.

The study hypotheses were that: 1) the intervention group, in comparison with the active control group, will have a larger increase of HRV; and 2) the intervention group, in comparison with the active control group, will have a smaller increase in serum inflammatory markers.

## Methods

### Sample

**Fig. 1:**
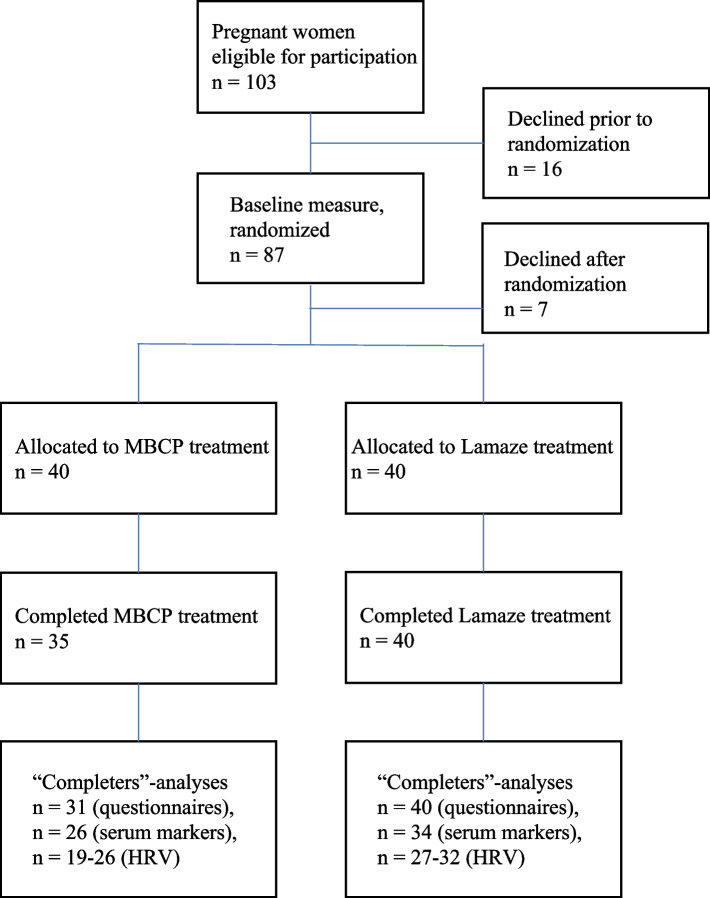
Flowchart of participants invited, screened, enrolled, and completing the study (CONSORT figure)

This study is a sub-study of a larger RCT, where perceived stress (assessed with the Perceived Stress Scale) was the primary outcome [34]. The Stockholm Regional Ethics Committee approved the study (approval number 2012/400-31/4) and written informed consent was obtained from all participants. Participants were blinded to the study hypothesis and recruited through eight maternity health clinics (MHCs) in Stockholm County between 2014 and 2015. The inclusion criteria were being a woman with a history of depression and/or anxiety and/or early life adversity and/or current high levels of perceived stress –in order to capture a sample of women who are at increased risk to develop PND. The details of study recruitment, procedures and eligibility have been described in detail in another publication [34]. For the larger RCT that this study is part of, a letter of invitation was sent to 1647 primiparous women. Of those, 347 women were assessed for eligibility and 193 met the inclusion criteria and agreed to participate; 96 were randomized to the MBCP group and 97 to the Lamaze group, see below. Blood samples and HRV measures for the present study were intended to be collected from the first 80 participants that were included in the study (40 in the intervention and 40 in the control group). However, all data was not available for all participants, due to various reasons. Hence, the N for each outcome variable is different - for further information on participation, see flowchart (Fig. 1). Trial registration: ClinicalTrials.gov ID: NCT02441595.

### Procedure

Eligible participants were scheduled for an appointment at the MHC for HRV-registration and the baseline questionnaires were completed via an on-line questionnaire that was sent to them via e-mail. Also, participants were referred to a health care centre to leave their blood samples before randomization – they informed the research team via e-mail once they had left the blood sample. After baseline assessments, an independent administrator randomised the participants to either MBCP or Lamaze, according to a sequence generated in SPSS in blocks of 10. MBCP participants joined the program within two weeks after baseline assessment, and Lamaze participants joined this program at between three and five weeks after baseline assessment. This strategy was applied as the Lamaze program was shorter than the MBCP program. At ten to twelve weeks after the baseline assessment both groups completed post-intervention assessments.

### Intervention

The original 9-week MBCP program was developed in the USA and consists of nine 3-hour long weekly sessions, a full day retreat and a reunion [6]. In the current trial we adjusted the curriculum to Swedish conditions – see Table 1 for details.

**Table 1:** Overview over the MBCP–curriculum adapted for the present study. Session Theme and practices [34]

1 Introduction to mindfulness and introduction of the teacher and the participants. Practice: mindfully eating a raisin and body scan.
2 Mind-body perspectives of childbirth regarding pain and fear, stress-hormones and the role of oxytocin and endorphins. Practice: body scan.
3 Coping with pain. Information about medical and non-medical analgesics. Practice: mindful yoga and pain-practice holding ice-cubes and exploring how pain and time is experienced differently depending on how and where one pays attention.
4 The role of the partner and how to best support a woman in labour. Practice: sitting meditation and pain-practice in couples supporting each other while holding one hand in ice water.
5 The needs of a newborn and new parents, secure attachment and child-development. Practice: sitting meditation and reflection on one's own childhood and expectations of parenthood and gender-roles.
6 Mindful communication. Practice: sitting meditation, lovingkindness meditation and interpersonal mindful speaking and listening inquiry reflecting on fear and joy.
7 Breastfeeding and the mind/body connection regarding prolactin, oxytocin, the let-down reflex and stress/anxiety versus calmness. Practice: sitting meditation.
8 Review of the course. Encouragement to continue practicing mindfulness, especially informal meditation with the baby after the birth. Practice: body scan.

### Active control

In order to control for the effects of social support, psychoeducation and child-birth preparation, the active control condition consisted of a Lamaze program that is widely available in Stockholm (for more details see [34]).

#### Outcome measures

In the present study, we used self-report questionnaire data from the same participants that we collected HRV and serum markers from, to allow for correlation analyses of these outcomes. The following questionnaires were used:

- **Perceived Stress Scale (PSS).** The PSS contains fourteen items that are used to assess perceived stressful experiences [11]. A Swedish version has been translated and validated [20].
- **Edinburgh Postnatal Depression Scale (EPDS).** The EPDS contains ten items that are used to assess depressive symptoms [12, 32], and a Swedish version has been validated [43].
- **Five-facet Mindfulness Questionnaire (FFMQ).** The validated Swedish Version of the FFMQ contains 29 items that are used to assess five facets of mindfulness [5, 33].

In the larger RCT of which this study is a part, we assessed internal consistency for all three scales at baseline (Cronbach alpha for PSS = .82, for EPDS = .85, for FFMQ = .85 and FFMQ subscales = .82, .75, .84, .88, .84).

#### Serum markers

Prior to randomisation, at pregnancy week 20-25, as well as at post-intervention, at pregnancy week 30-35, the participants left blood samples at a health clinic laboratory. 5 ml of blood was collected from each participant and then left to cool down for 30-60 minutes in room temperature before being centrifuged for 10 minutes at 2400 x gravity. The serum from each tube was thereafter aliquoted into two separate tubes, and frozen at -20°C. The biological outcomes in this study included serum levels of Interleukin-10 (IL-10), Interleukin-6 (IL-6), Interleukin-1β (IL-1β), Tumor Necrosis Factor-α (TNF-α), osteocalcin and nine acute phase proteins (alpha-2-microglobulin (A2M), C-reactive protein (CRP), haptoglobin, serum amyloid P (SAP), procalcitonin (PCT), ferritin, tissue plasminogen activator (tPA), fibrinogen and serum amyloid A (SAA)). Serum levels were assessed by using a premixed, magnetic bead-based multiplex panel (BioRad, Hercules, CA, USA), according to the manufacturer instructions. Concentrations were imputed using Bio-Plex 200 Suspension Array System with Bio-Plex Manager 6.0 software (Hercules, CA, USA). Coefficients of variation were calculated for the manufacturer-provided analytical controls. Samples below the lower level of quantification (LLOQ) were assigned the value of the LLOQ/**√**2 for the analyses.

#### Heart rate variability

ECG assessment was carried out at baseline and post-intervention, in a supine position on a stationary bench. Three ECG electrodes were placed over the left fifth intercostal space, the right fifth intercostal space and over the manubrium. Once the ECG signal was acceptable, participants were informed that they should rest for 10 minutes, alone in the examination room, before the ECG would be recorded for minimum 5 minutes. Participants were instructed not to talk or move excessively, to turn off their mobile phones, and to relax as much as possible. The ECG-assessments were recorded at a sampling frequency of 1000 Hz and stored on a computer. Each 300 s ECG-recording was inspected for ectopic beats and artifacts, as well as for the correct identification of each R-peak by the software, and non-sinus beats and other artifacts were corrected by interpolation [24]. After preparation of the data, time domain measures of HRV such as SDNN (Standard Deviation of the N-N intervals) and RMSSD (Root Mean Square of the Successive Differences), were computed, as well as power spectral analysis of the frequency domain, that partitions the total variance (the “power”) of a continuous series of beats into its frequency components: Low Frequency (LF), 0.04–0.15 Hz, High Frequency (HF) 0.15–0.4 Hz, and the ratio of LF to HF (LF/HF) [1, 2].

#### Statistical analyses

Independent sample t-tests were used for parametrically distributed data on interval scale level. For non-parametric data the Mann-Whitney U test was used to compare groups. Variables on nominal levels were tested by Fisher´s exact test. Some of the variables were skewed and therefore transformed to their natural logarithm (ln) before analyses with parametric tests could be conducted. Those analytes that had more than 10% of values with LLOQ were transformed into binary variables, where 0 denoted “under LLOQ” and 1 denoted “over LLOQ”. Spearman rank correlations (R_s_) were calculated to assess the correlations between the questionnaire target variables and blood sample target variables/HRV at baseline, and a Bonferroni correction was used to adjust for multiple comparisons. Within group comparisons from pre- to post-intervention were carried out using either paired t-tests for parametric distributions or Wilcoxon signed-rank test for non-parametric distributions. Linear regression analyses (or multinomial logistic regression analyses for categorical variables) were conducted to assess whether there were any significant differences between the intervention and control group in the change of target variables (post-intervention values minus pre-intervention values. Since the participating women entered the study (and hence, the pre-test) at different stages of pregnancy, the regression analyses were adjusted for pregnancy week since both serum inflammatory markers and HRV are considered to be related to gestation progress. The regression analyses for psychometric measures where not adjusted for pregnancy week, since pregnancy week was found not to be a confounder in the exploratory analyses. Only data from participants who completed the post-intervention assessments was analyzed. All analyses were performed using Stata software (version 14.2).

## Results

### Background of participants

Table 2 displays the descriptive statistics and group comparisons for demographic variables and shows a significant difference in pregnancy week at pre-test (*p*=0.0472). There were no significant baseline differences between groups in age, civil status, nationality, education, income or prescribed drug use.

**Table 2:** Socio-economic background characteristics of all participants (*n*=60)

Variable	**MBCP** **(*n* = 26)**	**Lamaze** **(*n* = 34)**	**Condition** **Comparisons**	***df***	***p***
Age, years					
*Mean*	31.6	32.9	*t* = -1.435	85	0.155
*SD*	3.93	4.70			
Civil status	n (%)	n (%)	*Fisher´s exact test*		1.000
Single	0 (0%)	1 (2%)			
Co-living	25 (60%)	26 (58%)			
Married	17 (40%)	17 (38%)			
Living apart	0 (0%)	1 (2%)			
Nationality			*Fisher´s exact test*		0.814
Swedish	37 (88%)	38 (84%)			
Swedish & other	2 (5%)	4 (9%)			
European	2 (5%)	1 (2%)			
Non-European	1 (2%)	2 (4%)			
Education			*Fisher´s exact test*		1.000
Elementary	0 (0%)	0 (0%)			
Secondary	7 (17%)	8 (18%)			
College	35 (83%)	37 (82%)			
Pregnancy week at pre-test					
*Mean*	24.3	22.9	z=1.98		0.0472
*SD*	3.85	3.77	*Mann-Whitney		
Household income/month			*Fisher´s exact test*		0.223
< 25 000 SEK	0 (0%)	0 (0%)			
25 - 40 000 SEK	5 (12%)	5 (11%)			
40 - 60 000 SEK	17 (40%)	11 (24%)			
> 60 000 SEK	20 (48%)	29 (64%)			
Prescribed drug use			*Fisher´s exact test*		0.377
None	25 (60%)	33 (73%)			
Other medication	12 (29%)	9 (20%)			
ADHD medication	0 (0%)	1 (2%)			
SSRI	4 (10%)	2 (4%)			
Sedatives	1 (2%)	0 (0%)			

*SD* Standard deviation, *SEK* Swedish crown, *ADHD* Attention deficit hyperactivity disorder, *SSRI* Selective serotonin reuptake inhibitor

### Baseline differences between groups in psychometric measures, serum markers and HRV

No significant baseline differences between groups in outcome variable values were found, apart from a-2 microglobulin and IL-6 where higher levels were found in the intervention group at baseline with *p*-value = 0.025 and 0.036 respectively.

### Correlations between psychometric measures and serum markers/HRV at baseline

There was no correlation found between any of the questionnaire target variables and blood sample target variables / HRV at baseline or at post-intervention (analysis performed for all participants together).

### Within group changes

Table 3 displays the within group changes in the psychometric measures between different time points.

**Table 3:** Within group changes, psychometric measures. Wilcoxon signed-rank test

**Outcome measures**	**MBCP** **(*n*=26)**				**Lamaze** **(*n*=34)**			
	**Baseline** **Mean (SD)** ***Median***	**Post-int** **Mean (SD)** ***Median***	***p*-value**	**Effect size (r)**	**Baseline** **Mean (SD)** ***Median***	**Post-int** **Mean (SD)** ***Median***	***p*-value**	**Effect** **size (r)**
PSS	27.774 (8.492) *28*	21.774 (6.835) *22*	≤ 0.001	- 0.68	25.275 (7.049) *26*	22.525 (9.047) *21*	0.0050	- 0.44
EPDS	9.967 (5.136) *10*	6.742 (3.812) *6*	0.0010	- 0.60	8.475 (5.179) *9*	7.425 (5.629) *7.5*	0.0484	- 0.31
FFMQ	87.200 (14.175) *86*	98.233 (12.266) *98*	≤ 0.001	0.80	93.425 (12.796) *91*	99.6 (14.836) *99*	≤ 0.001	0.59

*Abbreviations*: Perceived Stress Scale (*PSS*), Edinburgh Postnatal Depression Scale (*EPDS*), Five Facets Mindfulness Scale (*FFMQ*)

In the intervention group, the within-group comparisons of the psychometric variables from baseline to post-intervention showed significant decreases in perceived stress (PSS) scores (*p*≤0.001), and perinatal depression (EPDS) scores (*p*≤0.001) and significant increases in mindfulness (FFMQ) scores (*p*≤0.001).

In the control group, the within-group comparisons of the target variables from baseline to post-intervention showed a significant decrease in PSS scores (*p*=0.005) and EPDS scores (*p*=0.048) and significant increases in FFMQ scores (*p*≤0.001).

Table 4 displays the within group changes in the inflammatory serum markers between different time points.

**Table 4:** Within group changes, serum markers. ^1^=Paired t-test, ^2^=Wilcoxon signed-rank test

**Outcome measures**	**MBCP** **(*n*=26)**				**Lamaze** **(*n*=34)**			
	**Baseline** **Mean (SD)** ***Median***	**Post-int** **Mean (SD)** ***Median***	***p*-value**	**Effect size (Cohen´s d**^**1**^**/r**^**2**^**)**	**Baseline** **Mean (SD)** ***Median***	**Post-int** **Mean (SD)** ***Median***	***p*-value**	**Effect** **size (Cohen´s d**^**1**^**/r**^**2**^**)**
A2M^1^	99209 (113106) *75156*	145611 (196816) *48317*	0.299		60174 (65295) *41501*	88612 (158265) *36290*	0.138	
Hapto^1^	55286 (74169) *30459*	170919 (418747) *24428*	0.0412	0.30	29816 (36033) *19814*	77298 (159572) *24015*	0.0173	0.35
CRP^2^		N/A	0.564		N/A	N/A	0.527	
SAP^2^	1684 (1912) *1153*	2970 (3269) *1532*	0.0319	0.42	1189 (1302) *711*	2075 (2922) *910*	0.0428	0.35
PCT^1^	10989 (4555) *9586*	10877 (5190) *10120*	0.673		12657 (6710) *10730*	13602 (6866) *12473*	0.141	
Ferr^1^	37687 (57439) *14300*	25077 (20970) *22368*	0.796		45330 (62636) *18732*	35411 (46080) *24513*	0.576	
tPa^2^	5162 (4302) *3346*	8419 (8161) *7142*	0.0124	0.49	6935 (4998) *3939*	8934 (5481) *9505*	≤ 0.001	0.59
Fibri^1^	5970 (2034) *5198*	6442 (2584) *5782*	0.227		6920 (2398) *5948*	7544 (2567) *7324*	0.0220	
SAA^1^	1477 (1189) *953*	1673 (1312) *1326*	0.262		2330 (2051) *1478*	2652 (2476) *1591*	0.161	
Osteo^2^	1606 (1197) *2286*	2872 (2434) *1641*	≤ 0.001	0.79	1338 (1208) *977*	1991 (1942) *1128*	≤ 0.001	0.71
IL-1β^2^	N/A	N/A	0.317		N/A	N/A	1.0000	
IL-6^2^	N/A	N/A	0.480		N/A	N/A	0.317	
IL-10^2^	N/A	N/A	N/A		N/A	N/A	0.157	
TNF- α^2^	N/A	N/A	0.706		N/A	N/A	0.480	

N/A indicates “not applicable”, since these makers were dichotomized and mean values were thus not calculated

*Abbreviations*: Interleukin-10 (*IL-10*), Interleukin-6 (*IL-6*), Interleukin-1β (*IL-1β*), Tumor Necrosis Factor-α (*TNF-α*), Alpha-2-microglobulin (*A2M*), C-reactive protein (*CRP*), Serum amyloid P (*SAP*), Procalcitonin (*PCT*), Tissue plasminogen activator (*tPA*), Serum amyloid A (*SAA*)

In the intervention group, the within-group comparisons of the serum variables from baseline to post-intervention showed significant increases in serum levels of haptoglobin (*p*=0.041), serum amyloid P (*p*=0.032), tissue plasminogen activator (*p*=0.012) and osteocalcin (*p*≤0.001).

In the control group, significant increases from baseline to post-intervention serum levels of haptoglobin (*p*=0.017), serum amyloid P (*p*=0.043), tissue plasminogen activator (*p*≤0.001), fibrinogen (*p*=0.022) and osteocalcin (*p*≤0.001) were found.

No HRV parameters showed significant changes from baseline to post-intervention, neither in intervention- nor in control group (data not shown).

### Between group comparisons

Table 5 displays the differences between the intervention and control group in the change of target variables (post-intervention values minus pre-intervention values). The MBCP group reported a larger reduction in perceived stress (*F*(1, 69)= 4.54, adj *R*^2^=0.0481, *p*=0.037) and a larger increase in five facets of mindfulness (*F*(1, 69)= 4.52, adj *R*^2^=0.0485, *p*=0.037). No other target variables showed significant differences between the intervention and control group.

**Table 5:** Between group changes, (post-intervention values minus pre-intervention values). ^1^=linear regression, ^2^=multinomial regression

**Outcome measures**	**F**	**Adjusted R-squared**	**Multinomial logistic regression coefficient (group)**	**Multinomial logistic regression coefficient (preg week)**	***p*-value (group)**	**Effect size (Cohen´s f)**	***p*-value (preg week)**
**Serum markers (*n*=46)**	**F (2, 57)**						
A2M^1^	0.18	-0.0284			0.605		0.695
Hapto^1^	0.99	-0.0005			0.429		0.315
CRP^2^			1.515	0.299	0.107		0.034
SAP^1^	0.43	-0.0195			0.610		0.496
PCT^1^	2.54	0.0495			0.495		0.050
Ferr^1^	0.26	-0.0256			0.756		0.488
tPa^1^	0.47	-0.0183			0.340		0.780
Fibri^1^	1.46	0.0154			0.914		0.101
SAA^1^	1.74	0.0246			0.933		0.073
Osteo^1^	6.55	0.1582			0.160		0.003
IL-1β^2^			1.034	-0.238	0.428		0.040
IL-6^2^			-1.735	-0.101	0.152		0.365
IL-10^2^			16.906	0.361	0.996		0.120
TNF- α^2^			0.204	0.00448	0.800		0.966
**HRV**							
SDNN^1^ (*n*=56) F (2, 53)	0.69	-0.0114			0.757		0.249
RMSS^1^ (*n*=49) F(2, 46)	0.74	-0.0108			0.288		0.481
LFpow^1^ (*n*=52) F(2, 49)	0.94	-0.0025			0.190		0.634
LFnorm^1^ (*n*=58) F(2, 55)	2.06	0.0358			0.805		0.060
HFpow^1^ (*n*=49) F(2, 46)	1.12	0.0051			0.748		0.169
HFnorm^1^ (*n*=56) F(2, 53)	2.10	0.0386			0.505		0.080
LF/HFpow^1^ (*n*=46) F(2, 43)	0.35	-0.0297			0.951		0.410
**Questionnaires** **(*n*=71)**	**F (1, 69)**						
PSS^1^	4.54	0.0481			0.037	0.22	N/A
EPDS^1^	3.92	0.0406			0.052		N/A
FFMQ^1^	4.52	0.0485			0.037	0.23	N/A

N/A indicates “not applicable”, since these analyses were not adjusted for pregnancy week

*Abbreviations*: Interleukin-10 (*IL-10*), Interleukin-6 (*IL-6*), Interleukin-1β (*IL-1β*), Tumor Necrosis Factor-α (*TNF-α*), Alpha-2-microglobulin (*A2M*), C-reactive protein (*CRP*), Serum amyloid P (*SAP*), Procalcitonin (*PCT*), Tissue plasminogen activator (*tPA*) and serum amyloid A (*SAA*), Standard Deviation of the N-N intervals (*SDNN*), Root Mean Square of the Successive Differences (*RMSSD*), Low Frequency power (*LFpow*), Low Frequency normalized (*LFnorm*), High Frequency power (*HFpow*), High Frequency normalized (*HFnorm*), Perceived Stress Scale (*PSS*), Edinburgh Postnatal Depression Scale (*EPDS*), Five Facets Mindfulness Scale (*FFMQ*)

## Discussion

In this study, no significant changes in our primary outcomes, inflammatory serum markers or HRV-measures, were found.

However, in line with the results from the main study, participants randomized to MBCP reported a significantly larger reduction in perceived stress and a significantly larger increase in mindfulness, compared to participants randomized to Lamaze. The finding of significant differences between groups in reductions in PND [34] were not replicated in this sub-study. Nevertheless, the main purpose of this sub-study was not to assess between-group differences in changes in self-report measures.

This study is the first one to compare the effects of MBCP with an active control group not only on psychological outcomes, but also on physiological outcomes. The lack of significant effects of MBCP on inflammatory serum markers and HRV parameters found in this study are in line with the results reported in a recent review and meta-analysis [41].

A normal pregnancy is related to remarkable changes in the immune system. Complex adaptations are made to protect the fetus against pathogens as well as to adjust the mother´s immunological response in order to prevent rejection of the fetus [8, 29]. Due to this, the results from the current study on inflammatory serum markers can also be interpreted as a normal immunological shift that occurs in pregnancy and hence explain the lack of significant differences between MBCP and Lamaze. Both groups showed significant increases in haptoglobin, serum amyloid P, tissue plasminogen activator and osteocalcin, which can probably be explained by normal changes over time during pregnancy [8]. In addition to changes in the immune system, a normal pregnancy is also related to an increase in sympathetic tone as the gestation progresses [19, 23]. This may in turn result in altered HRV parameters [28, 47], which may have rendered eventual changes from the intervention undetectable in the present study.

The findings on larger reduction in perceived stress and larger increase in mindfulness in the MBCP group compared to the Lamaze group, are in line with previous findings [4]. However, the present study did not confirm our hypothesis that the MBCP program would incur greater positive impact on the stress-and depression-related physiological variables studied. This may be explained by that the control intervention - the Lamaze childbirth program - also has physiological impacts for the pregnant women, as has been demonstrated in a meta-analysis [50].

### Limitations of the study

This study has both limitations and strengths. A strength is the randomization of participants and the use of an active control intervention. However, we were not able to conduct a sample size calculation for the present study, due to the scarcity of other studies in this research field. Therefore, we cannot be certain that the lack of detectable differences between the groups in terms of biomarkers were due to the lack of actual changes in HRV and inflammatory parameters, or due to lack of statistical power. Due to the large number of statistical analyses conducted in the study, significant changes in certain variables should be interpreted with caution.

### Future research

In this study, it was not possible to assess if some of the changes that were detected in biomarkers over time were due to the interventions or if they were naturally occurring during pregnancy. Therefore, it may be of interest to conduct future studies where a third arm with no intervention is included. Also, future studies may need to include larger samples, and the present study findings may be used as a basis for a future sample size calculation. Based on for example the CRP outcome in this study, we have been able to conduct a post hoc power calculation (4% change in intervention group vs. 0% change in control group), which gives 24.4% power.

## Conclusion

Our results suggest that MBCP is more effective in decreasing perceived stress and increasing levels of mindfulness, compared to a Lamaze childbirth program. However, no significant differences between MBCP and the Lamaze program were found regarding changes in HRV measures and biomarkers of inflammation.

## Data Availability

The datasets used and/or analysed during the current study are available from the corresponding author on reasonable request.

## Abbreviations

PND: Perinatal depression
MBI: Mindfulness Based Interventions
MBSR: Mindfulness Based Stress Reduction
HRV: Heart Rate Variability
MBCP: Mindfulness Based Childbirth and Parenting
PSS: Perceived Stress Scale
EPDS: Edinburgh Postnatal Depression Scale
FFMQ: Five Facets Mindfulness Scale
IL-10: Interleukin-10
IL-6: Interleukin-6 (IL-6)
IL-1β: Interleukin-1β
TNF-α: Tumor Necrosis Factor-α
A2M: Alpha-2-microglobulin
CRP: C-reactive protein
SAP: Serum amyloid P
PCT: Procalcitonin
tPA: Tissue plasminogen activator
SAA: Serum amyloid A
SDNN: Standard Deviation of the N-N intervals
RMSSD: Root Mean Square of the Successive Differences
LFpow: Low Frequency power
LFnorm: Low Frequency normalized
HFpow: High Frequency power
HFnorm: High Frequency normalized
